# A Patent Foramen Ovale With an Atrial Septal Aneurysm in a Patient Presenting With Deep Vein Thrombosis and Pulmonary Embolism

**DOI:** 10.7759/cureus.53714

**Published:** 2024-02-06

**Authors:** Gabriel Panama, Adolfo Martinez, Saif Alattal, Preeti Banga, Sandeep Banga, Mohammed Quintar

**Affiliations:** 1 Internal Medicine, Michigan State University, East Lansing, USA; 2 Radiology, Michigan State University, East Lansing, USA; 3 Cardiology, Michigan State University, East Lansing, USA; 4 Interventional Cardiology, Sparrow Hospital, Lansing, USA

**Keywords:** massive pulmonary embolism, deep vein thrombosis (dvt), patent foramen ovale closure, patent foramen ovale (pfo), atrial septal aneurysm

## Abstract

Patent foramen ovale (PFO) is an embryogenic remnant that can be found in healthy adults with no repercussions. However, it poses a risk of paradoxical embolism. In patients with known embolic stroke, the risk of recurrence is greater. A PFO can be accompanied by morphological variants such as atrial septal aneurysms (ASA). These have been shown to further increase the risk of stroke and embolism. This is a case of a patient who presented to the emergency department with deep vein thrombosis and sub-massive pulmonary embolism. An echocardiogram showed a PFO with an ASA as an incidental finding. The defect was closed with a transcatheter PFO closure device due to a high risk of paradoxical embolism.

## Introduction

The foramen ovale is a communication between the atria that exists during fetal life to allow the flow of oxygenated blood from the placenta to the systemic circulation. After birth, the foramen ovale should close by 12 months; beyond this, is considered a patent foramen ovale (PFO).

Having a PFO confers a risk of having a cryptogenic stroke (CS) by causing a paradoxical embolism. Up to 25% of the general population can have a PFO, but not all PFOs lead to a stroke [1]. The risk of having a stroke in healthy adults with a PFO is 0.1% per year. However, a person with a history of stroke due to a PFO has a risk of recurrence of embolic stroke of approximately 3.4%-11% [2].

PFOs with certain morphological characteristics confer a higher risk of paradoxical embolism; hence, identifying these features can help in decision-making. These features include atrial septal aneurysm (ASA), right-to-left shunt (RLS), PFO tunnel length > 10 mm, low-angle PFO, large-size PFO with height > 2 mm, presence of a prominent Eustachian valve, and Chiari network [2].

Identifying PFOs with high-risk morphological features is of great significance as these are associated with a higher risk of stroke and affect procedural outcomes. In this case, we examine the role of ASAs in PFOs.

## Case presentation

A 67-year-old male presented to the emergency department complaining of left calf pain associated with shortness of breath, pleuritic chest pain, and hemoptysis. He denied recent prolonged immobilization, surgery, or active cancer. On admission, the patient was vitally stable with normal oxygen saturation on room air.

**Figure 1:**
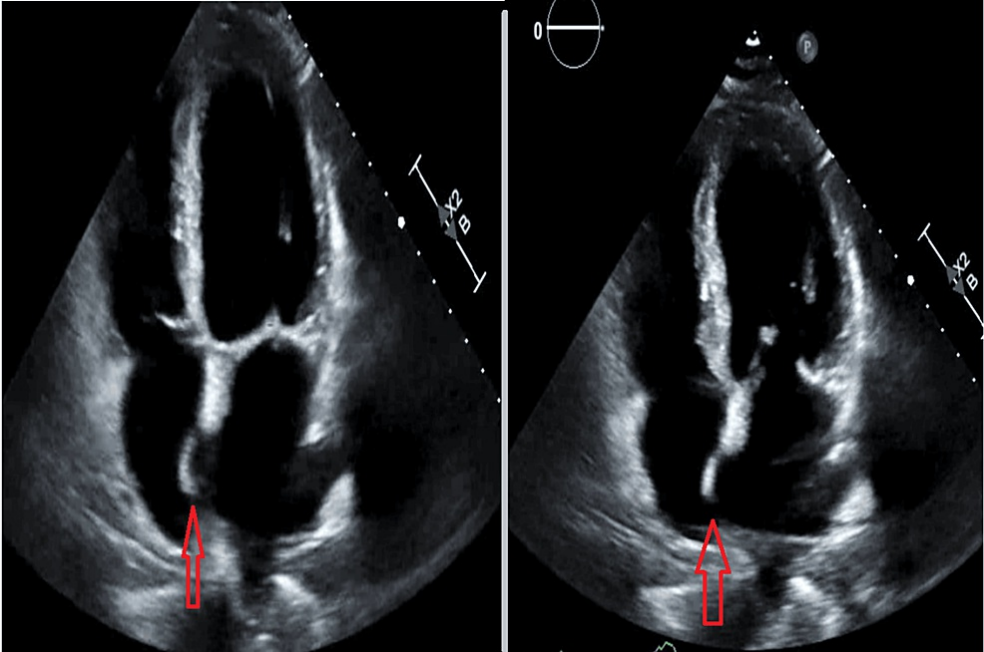
TTE (apical four-chamber view). Arrows showing excursion of the atrial septum into the right atrium. This is compatible with an atrial septal aneurysm. TTE, transthoracic echocardiography

Imaging showed a left popliteal and superficial femoral deep vein thrombosis (DVT), and a CT scan was remarkable for severe pulmonary embolus in the left pulmonary artery with saddle embolus and emboli in the right lower and upper lobe arteries. Transthoracic echocardiography (TTE) showed a left ventricular ejection fraction of 50-55%. A severely dilated right ventricle with strain and a D septum was also noted. A PFO with an ASA and RLS was noted (Figure 1; Video 1). A Bubble study was positive (Video 2). Lower left extremity ultrasound showed a superficial femoral and popliteal venous thrombus.

**Video 1:** TTE (apical four-chamber view). Atrial septal excursion into both atria can be appreciated. This is compatible with an atrial septal aneurysm. TTE, transthoracic echocardiography

**Video 2:** TTE (bubble study). Appreciate how the bubbles first fill the right heart chambers, and then bubbles are seen in the left heart chambers. This is positive for a patent foramen ovale. TTE, transthoracic echocardiography

**Figure 2:**
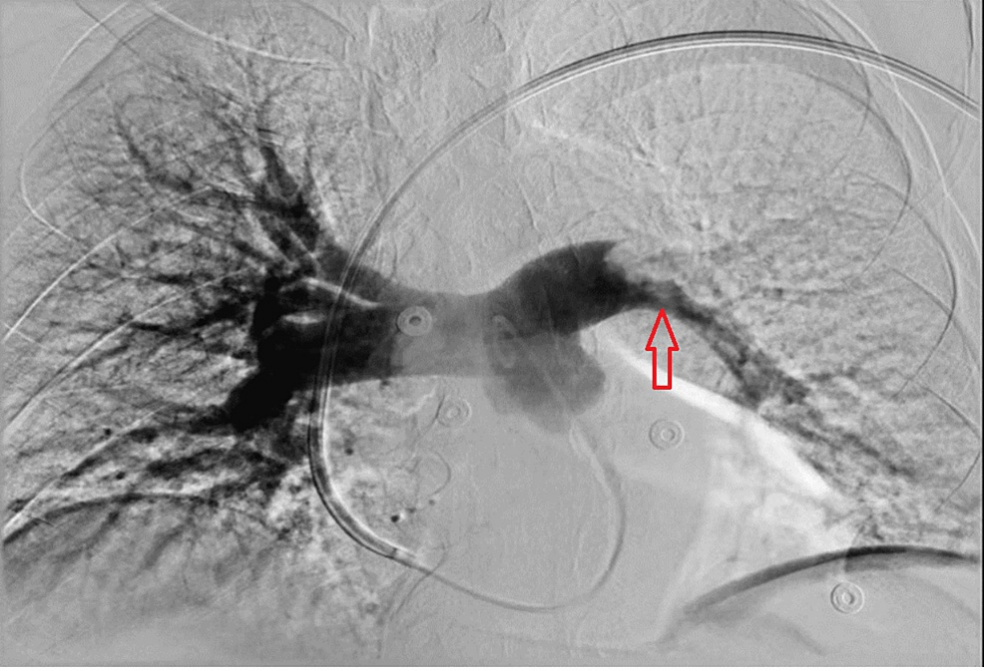
Invasive pulmonary angiogram. Arrow shows filling defect in the left pulmonary artery. This is consistent with a pulmonary embolus.

**Figure 3:**
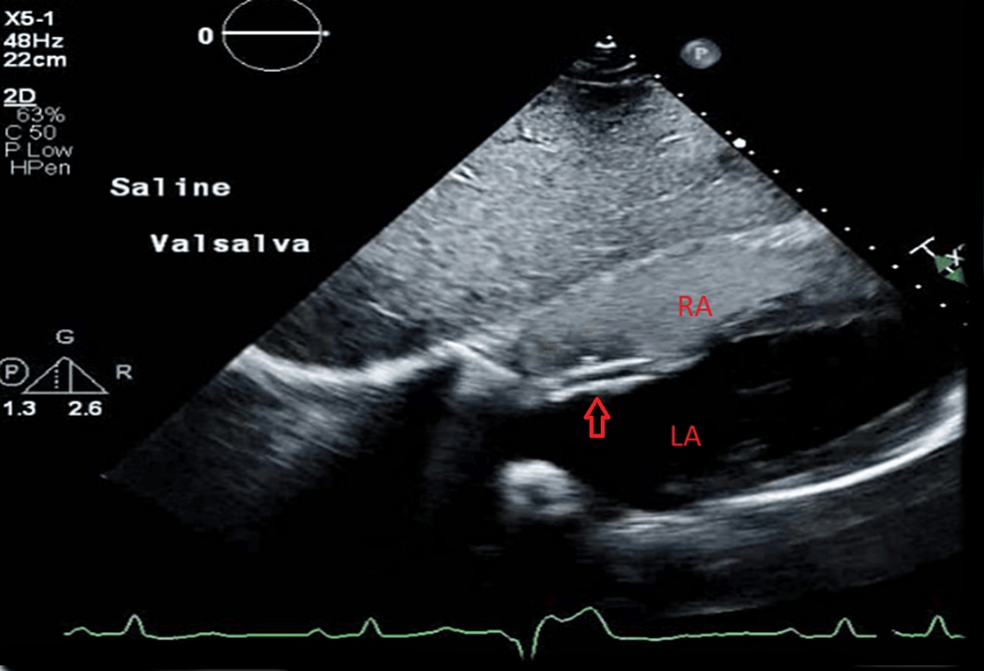
Echocardiography showing closure of the patent foramen ovale. Arrow shows the Amplatzer device in place. Note that there is no passage of bubbles from RA to LA. RA, right atrium; LA, left atrium

The patient was started on a heparin drip, and an inferior vena cava (IVC) filter was placed. Due to a high risk of paradoxical embolization, he was taken to the Cath lab where the ASA was closed using an Abbott 30-mm cribriform Amplatzer device. Thrombectomy was unsuccessful due to severe angulation and dilation of the right ventricle (Figure 2). Post-procedural echocardiography showed placement of the device in the interatrial septum, with improvement in right ventricular dilation and systolic function (Figure 3).

The patient was discharged on Eliquis and aspirin, and it was recommended to get the IVC filter removed in one to two weeks after discharge.

## Discussion

ASAs are defined by the American Society of Echocardiography as an excursion of the atrial septum into the atrium >10 mm or a total excursion of >15 mm [1]. They are found concomitantly with structural abnormalities such as PFO in 44% of cases, in addition to valvulopathies such as mitral and aortic regurgitation [1]. They are associated with arrythmias, including paroxysmal atrial fibrillation, supraventricular tachycardia, and premature ventricular complexes. There is also an association with migraines [3].

ASA confers thrombotic risk through different proposed mechanisms. The aneurysmal sac causes left atrial dysfunction by decreasing active and passive emptying, leading to blood stasis and thrombus formation within the sac [4]. ASAs also increase the prevalence of atrial fibrillation. The combination of ASA and a PFO has been shown to increase the risk of stroke [5].

Echocardiography is the mainstay imaging modality used for assessing ASAs. Transesophageal echocardiogram (TEE) is superior to transthoracic echocardiography (TTE) in detecting ASAs. It provides a better characterization of the morphology and estimation of the size of the septal defect. It also has a higher sensitivity and specificity for detecting PFOs through a bubble study [1]. TEE including its 3D modality can be used to guide intraprocedural closure of the defect [6,7].

Since up to 25% of healthy adults can have a PFO, associating it as the cause of a stroke can be challenging [1]. The Risk of Paradoxical Embolism (RoPE) score is a scoring system developed to identify a PFO as the cause. It entails demographics such as patient age, prior vascular risk factors, and brain imaging [8]. However, this scoring system does not consider the morphological characteristics of a PFO. Nakayama et al. found that high-risk features such as a long-tunnel PFO >10 mm, hypermobile atrial septum, eustachian valve, Chiari’s network, large RLS during Valsalva maneuver, and low-angle PFO were independent of CS. They created a 4-point scoring system with each feature allocated a value of 1 point. A score equal to 2 or more had an 80% risk of CS [9].

In the REDUCE and CLOSE trials, evidence suggested that transcatheter closure with a device was superior at preventing recurrent embolic stroke compared to medical therapy (antiplatelet therapy or anticoagulation). The DEFENSE-PFO trial in particular only enrolled patients with high-risk PFO features defined as a PFO with an ASA, septal hypermobility, and PFO size ≥ 2 mm [8,10]. Its findings were consistent with the previous studies mentioned.

Septal morphology can also affect transcatheter closure in many ways. Von Bardeleben et al. analyzed three types of occluding systems - Amplatzer, Helex, and Starflex - followed by their closure rate with serial echocardiography in patients with PFO and PFO+ASA. Patients who had an ASA had lower closure rates at 6-month and 1-year follow-up [11]. In a study conducted using the Amplatzer occluder only, patients with ASA had higher rates of residual shunts after closure. It was also seen more with the 35 mm size compared to the 25 mm size [12].

## Conclusions

It is imperative to identify PFOs with high-risk features as this can alter the prognosis and treatment of a patient. Identifying a PFO as the culprit of a stroke can be challenging. Using risk-stratification tools that include high-risk features can help in better decision-making. Studies have shown that using a transcatheter device for closure is superior to medical treatment in preventing the recurrence of embolic stroke in both PFOs with and without high-risk features. ASAs can affect procedural outcomes by delaying the closure of the PFO after device closure.
